# Navigating the social world: The role of social competence, peer victimisation and friendship quality in the development of social anxiety in childhood

**DOI:** 10.1016/j.janxdis.2018.09.002

**Published:** 2018-12

**Authors:** Hannah Pickard, Francesca Happé, William Mandy

**Affiliations:** a Medical Research Council (MRC) Social, Genetic and Developmental Psychiatry (SGDP) Centre, King’s College London, Denmark Hill, London, SE5 8AF, UK; b Research Department of Clinical, Educational and Health Psychology, University College London, Torrington Place, London, WC1E 6BT, UK

**Keywords:** Social and communication difficulties, Social anxiety, Longitudinal, ALSPAC

## Abstract

Social and communication (SC) difficulties predict increased social anxiety (SA) symptoms in childhood. Peer victimisation and friendship quality are commonly associated with both SC difficulties and SA. Based on this, we tested for a cascade effect of early SC difficulties, peer victimisation and friendship quality on SA in late childhood, using a population-based sample of 8028 children from the Avon Longitudinal Study of Parents and Children (ALSPAC) cohort. Parent-reported data were collected on SC difficulties at age 7 and SA at age 7, 10 and 13. Child-reported data on peer victimisation and friendship quality were collected at age 8. Our results revealed that SC difficulties predict increased negative friendship qualities and peer victimisation. Relational victimisation predicted increased SA symptoms at 13 years old. Neither overt nor relational victimisation mediated the developmental relationship between SC difficulties and SA. Furthermore, friendship quality did not moderate the developmental relationship between SC difficulties and SA. In addition, no sex differences were observed. The evidence demonstrates that peer victimisation and friendship quality do not explain why some children with SC difficulties go on to develop SA. Future research clarifying the complex etiological pathways contributing towards the development of SA in childhood and adolescence is warranted.

## Highlights

• SC difficulties predict peer victimisation and negative friendship quality.
• Victimisation does not mediate the link between SC difficulties and social anxiety.
• Friendship quality does not interact with SC difficulties to predict social anxiety.
• Research exploring etiological pathways to social anxiety in childhood is needed.

## 1 Introduction

Social anxiety (SA) is a common experience, which lies on a continuum of severity in the general population (Knappe et al., 2011; Rapee & Spence, 2004). Severe symptoms of SA include a persistent intense fear of social situations in which a person may be scrutinised or negatively evaluated by others (American Psychiatric Association, 2013). SA is the third most common psychiatric disorder, with lifetime prevalence between 7–13% (Kessler, Petukhova, Sampson, Zaslavsky, & Wittchen, 2012). Although the typical age of onset occurs during early adolescence (Grant et al., 2005), clinically anxious pre-adolescent children are frequently diagnosed with social anxiety disorder (SAD; Costello, Egger, & Angold, 2005). Without effective treatment, childhood SA typically runs a chronic course, with a reduced likelihood of total remission and the risk of developing additional psychiatric disorders in adolescence (Bittner et al., 2007). Given the chronicity of SA and the impact on functioning and wellbeing across the life span, there is a need for longitudinal research to investigate mechanisms that underlie the development of SA across childhood, which will inform the development of targeted interventions to decrease a child’s risk of developing SAD.

Etiological models of SA in childhood and adolescence have implicated the role of several risk factors, including social skill deficits and negative peer relationships, among others (Spence & Rapee, 2016). One risk factor suggested to underpin the development of SA is social and communication (SC) difficulties. SC ability is considered to be a continuously distributed trait that is expressed to varying degrees in the general population, with some individuals exhibiting no SC difficulties and others at the extreme end exhibiting severe SC difficulties (Ronald & Hoekstra, 2011), often resulting in a diagnosis of autism spectrum disorder (ASD), which is also characterised by restricted and repetitive interests and behaviours. The prevalence of SA disorder is elevated amongst children with ASD and those with high autistic traits (Hallett et al., 2013; Salazar et al., 2015), which is indicative of a developmental link between SC difficulties and SA. This link has been supported by both cross-sectional and recent longitudinal research.

Cross-sectional research shows that children diagnosed with SAD exhibit more SC difficulties, as reported by observer, parent and self-ratings, compared to non-anxious children (Beidel, Turner, & Morris, 1999; Spence, Donovan, & Brechman-Toussaint, 1999) and children with other anxiety disorders (Halls, Cooper, & Creswell, 2015). Although the universality of SC difficulties in SA is debatable (Cartwright-Hatton, Tschernitz, & Gomersall, 2005), the evidence implies that for some children, SC difficulties are a risk factor for SA. Research in population-based samples characterised by a degree of SC difficulties has also provided consistent support. Using parent-report measures, Hallett and colleagues (2013) found that more ASD-like SC difficulties were associated with greater SA symptoms and higher IQ, implying that these relationships are consistent at a trait-wise level (Hallett et al., 2013). Limited by cross-sectional research designs, the question of whether SC difficulties contribute towards SA, or are a consequence of SA remains unanswered. Extending cross-sectional findings, a recent longitudinal study using a population-based sample of children (age 7–13 years) found a directional and specific relationship between SC difficulties and SA, with earlier SC difficulties modestly predicting later SA symptoms (Pickard, Rijsdijk, Happé, & Mandy, 2017). Importantly, the reverse relationship was not observed. These findings highlight that SC difficulties are a risk factor for SA in childhood. Further research identifying mechanisms that influence this developmental pathway to SA, such as negative peer relationships, is required to inform preventative interventions that aim to decrease a child with SC difficulties risk of developing later SA.

Peer victimisation and poor friendship quality, are proposed to play a role in the development of SA. Peer victimisation is a multifaceted phenomenon, which encompasses both overt (e.g. hitting, kicking) and relational victimisation (e.g. spreading rumours). Children who experience both subthreshold and clinical SC difficulties, including young people with ASD, often experience high rates of victimisation and peers problems (Cappadocia, Weiss, & Pepler, 2012; Erath, Flanagan, & Bierman, 2007; Skuse et al., 2009). In line with cross-sectional research, prospective research indicates that social skill problems predict both later peer victimisation and social isolation in typically developing children (Fox & Boulton, 2006). Several cross-sectional and prospective studies have reported associations between peer victimisation (Hawker & Boulton, 2000), in particular relational victimisation, and SA across childhood and adolescence (Dempsey, Sulkowski, Nichols, & Storch, 2009; La Greca & Harrison, 2005; Siegel, La Greca, & Harrison, 2009). Sex differences in the relationship between relational and overt victimisation and SA symptoms have frequently been reported. In adolescent girls, relational victimisation, but not overt victimisation, has been seen to predict an increase in later SA symptoms (Siegel et al., 2009). Of note, SA symptoms contributed towards increased relational victimisation for both boys and girls. A longitudinal study using a large sample of adolescents (age 15–17 years) found a bi-directional relationship between overt victimisation and SA symptoms in males, however, among females, only relational victimisation contributed towards later SA symptoms (Ranta, Kaltiala-Heino, Fröjd, & Marttunen, 2013). Mixed findings with regards to sex differences have often been reported. To date, limited research has examined the role of relational and overt victimisation, as well as sex differences, in predicting SA in pre-adolescent children. Developmental differences in the relationship between relational and overt victimisation and anxiety have been reported (Casper & Card, 2017), highlighting the need for research to elucidate the prospective relationships between peer victimisation and SA during earlier time points in development. In addition, as SC difficulties and victimisation are linked to the development of SA, further prospective longitudinal research using large population-based samples is warranted to explore the combined effect of SC difficulties and victimisation on SA throughout childhood.

Friendships are important experiences for developing social and cognitive skills across childhood and adolescence (Bagwell, Newcomb, & Bukowski, 1998). Experiencing SC difficulties during childhood can create barriers to forming positive friendships (Erath et al., 2007). Specifically, in anxious and non-anxious children (age 8–14), social skill problems have been linked to having a smaller number of best-friends and poor friendship qualities, which often refers to the quality of support and companionship experienced amongst peers (Crawford & Manassis, 2011; Fox & Boulton, 2006). Similar findings are observed amongst adolescents with severe SC difficulties, including individuals with ASD, who report both poor friendship qualities and lower social network status (Locke, Ishijima, Kasari, & London, 2010). In addition, experiencing negative friendships (e.g. poor quality, small number of friends/best-friend) is proposed to contribute towards the development of SA in childhood. Research indicates that youths with higher SA symptoms often have fewer friends, feel less accepted and liked by their peers, experience more negative friendship quality and more negative peer interactions (Blöte & Westenberg, 2007; Erath et al., 2007; La Greca & Lopez, 1998). Additionally, children with anxiety disorders who have higher symptoms of SA show lower peer liking, acceptance and more negative interactions with friends (Ginsburg, La Greca, & Silverman, 1998; Verduin & Kendall, 2008). To date, research supports the link between negative friendships and SA symptoms in childhood; however, no longitudinal research has explored the prospective relationships between friendship quality and SA in pre-adolescent children. Furthermore, to the author’s knowledge, no prospective research using population-based samples has explored whether friendship quality exacerbates the developmental relationship between SC difficulties and SA in childhood.

Etiological models of SA in normative development and neurodevelopmental disorders propose that, when experienced together, SC difficulties and negative peer relationships will exacerbate a youth’s risk of experiencing SA (Bellini, 2006; Spence & Rapee, 2016). For example, a child with SC difficulties may struggle to form stable friendships and as a consequence may be subjected to peer victimisation, which combined could act to reinforce withdrawal behaviours and lead to increased feelings of SA. Exploring these developmental pathways using longitudinal research designs may help us to clarify why some children with SC difficulties go on to develop SA, while others do not. To date, no longitudinal research has investigated whether peer victimisation and friendship quality influence the developmental pathway from SC difficulties to SA in childhood. This research will enhance our understanding of the risk factors that contribute towards the development of SA in childhood and will inform early target-specific preventative interventions for SA.

**Fig. 1:**
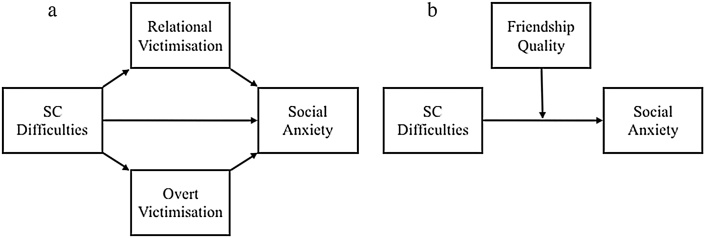
Conceptual diagram illustrating our (a) mediation and (b) moderation hypotheses. *Note.* SC = social and communication

In the present longitudinal study, we aim to examine whether negative friendship qualities, and overt and relational victimisation influence the developmental relationship between SC difficulties and SA symptoms using a population-based sample of children (see Fig. 1). In light of research signifying sex differences, the present study also aims to explore sex differences in our following hypotheses. We aim to test the following research questions: 1) Do SC difficulties lead to increased peer victimisation/negative friendship quality over middle/late childhood? 2) Does negative friendship quality/peer victimisation predict SA symptoms? 3) Does overt and/or relational victimisation mediate the relationship between SC difficulties and SA symptoms? 4) Does friendship quality moderate the relationship between SC difficulties and SA symptoms?

## 2 Method

### 2.1 Participants

The Avon Longitudinal Study of Parents and Children (ALSPAC) is a population-based cohort of children born in Bristol, UK, between 1991 and 1992. At one years old 13,988 children were alive and formed the original cohort (Boyd et al., 2013). Beginning in the first trimester of pregnancy parents completed postal questionnaires every year about themselves and their child’s development and health. In addition, children were invited to take part in clinic sessions. Please note that the study website contains details of all the data that is available through a fully searchable data dictionary http://www.bris.ac.uk/alspac/researchers/data-access/data-dictionary/. Ethical approval for the study was obtained from the ALSPAC Ethics and Law Committee and the Local Research Ethics Committees. The present study used children who participated in the age 7 data collection phase and had available SC difficulties data (N = 8028). Of those individuals, 6430 (80%) and 5784 (72%) participants had available SA data at age 10 and 13, respectively. Following ALSPACs exclusion criteria for prorated scores, only children with 50% or more complete data on SC difficulties at age 7 and SA at age 10 and 13 years were included in the present study. The final sample of 8028 (49% female, *n* = 3898) individuals was used in all final analyses. In the final sample, 42% of mothers were in higher education, 82% owned/mortgaged their own home and 96% were from a white ethnic background. Compared to those in the ALSPAC cohort who were not included in the following analyses, the current sample were more likely to have a mother who had completed higher education (OR = 2.26, 95% CI = 2.08, 2.45), was a homeowner (OR = 2.89, 95% CI = 2.67, 3.13) and was from a white ethnic background (OR = 1.98, 95% CI = 1.68, 2.33).

### 2.2 Measures

#### 2.2.1 Social economic status (SES)

Maternal education at 32 weeks of gestation was used as a measure of SES. Previous research has shown that this is a valid indicator of SES in the ALSPAC cohort (Aguinis, Beaty, Boik, & Pierce, 2005). Mothers reported on their highest education achievement to date from 6 options: “none”, “CSE” (lowest basic education in UK), “vocational”, “O-level”, “A-levels” and “degree or above”, with higher scores indicating better maternal education and higher SES.

#### 2.2.2 IQ

Wechsler Intelligence Scale for Children-Third Edition (WISC-III; Wechsler, Golombok, & Rust, 1992) is a measure of IQ for children aged 6–16 years. The abbreviated version of the WISC, which includes 10 randomly selected subtests, was administered to children during the clinical data collection wave at 8 years old. In the present sample, 5913 (74%) children have a complete full-scale IQ score (Mean = 105.24, SD = 16.31, Range = 45–151).

#### 2.2.3 Social anxiety

Development and Wellbeing Assessment (DAWBA; Goodman, Ford, Richards, Gatward, & Meltzer, 2000) was administered as a parent-report questionnaire to capture child and adolescent psychopathology in accordance with the ICD-10 and DSM-IV diagnostic criterion. The DAWBA social fears (DAWBA-SF) subscale was used to capture the development of SA symptoms across childhood. For the DAWBA-SF, parents report whether their child has been afraid of six possible situations (e.g. “meeting new people” and “eating in front of others”) over the past month. Four possible responses were recorded: “no”, “a little”, “a lot” and “hasn’t done this in the last month”. Any responses of “hasn’t done this in the last month” were excluded, as this response does not adequately answer any of the six questions. A social fear total score was created by summing responses over all items to give a score ranging from 0-12. Higher scores indicate greater SA symptoms. The DAWBA-SF scale showed good internal reliability at 7 (a = .79), 10 (a = .79) and 13 (a = .80) years old.

#### 2.2.4 SC difficulties

Social Communication and Disorders Checklist (SCDC; Skuse, Mandy, & Scourfield, 2005) is a 12-item parent-report questionnaire that measures a child’s social functioning and behaviours (e.g. “does not pick up on body language” and “does not seem to understand social skills e.g. interrupts conversations constantly”) over the past 6 months. The questionnaire item response scale ranges from “not true” = 0 to “very often true” = 2, with a total score ranging between 0-24. Higher scores indicate more SC difficulties. The SCDC shows good sensitivity (.88) and specificity (.93) when distinguishing between those with ASD, other clinical disorders and those without any formal diagnosis (Skuse et al., 2005). Previous research in the ALSPAC cohort has shown that the SCDC has good construct validity and is a reliable measure of SC ability in the general population (Skuse et al., 2009). Furthermore, within this large population-based sample the SCDC is seen as a measure of SC traits that genetically overlap with ASD, supporting the validity of the SCDC as a measure of ASD-like SC difficulties (St Pourcain et al., 2014). The SCDC scale showed excellent internal reliability (a = .88).

#### 2.2.5 Friendship quality

The Cambridge Hormones and Moods Project Friendship questionnaire (Goodyer, Wright, & Altham, 1989) was used to assess friendship quality at 8 years old. Children were asked to complete 5 questions (e.g. “are you happy with the number of friends you’ve got?” and “do you talk to your friends about problems?”) with answers ranging from 0-3. Summing the answers for the 5 items gave a total score between 0–15, with 0 indicating a more positive friendship score. The scale showed a modest average inter-item correlation (*r* = .21).

#### 2.2.6 Peer victimisation

A modified version of the Bullying and Friendship Interview Schedule (Wolke, Woods, Stanford, & Schulz, 2001) was used to assess peer victimisation at 8 years old. Trained psychologists assessed the child’s involvement in both relational (e.g. “had lies/told nasty things about them”) and overt (e.g. “been hit/beaten up”) victimisation by peers over the past 6 months. Children reported whether they had experienced 9 types of peer victimisation, responding “not at all” = 0 to “very frequently” = 3. A total victimisation severity score can be derived by summing all items, producing a total score ranging from 0 to 25, which showed good internal reliability (a = .73). In the present sample, separate variables for overt victimisation (0–15, a = .64) and relational (0–12, a = .65) victimisation were used in all analyses. In our sample, the overt and relational victimisation observed total scores were moderately correlated (*r* = .41; girls *r* = .42; boys *r* = .42). A widely used ordinal victimisation variable was derived from the total victimisation severity score to illustrate the frequency of child victimisation in our sample, using the following cut-offs (Bowes, Joinson, Wolke, & Lewis, 2015; Stapinski et al., 2014). Children scoring 4 or more were categorised as severely victimised, children scoring 1–3 were categorised as moderately victimised and those scoring 0 were categorised as not victimised. Acceptable levels of inter-rater agreement have been reported between child self-reports of victimisation and parent/teacher reports (Schreier et al., 2009).

### 2.3 Statistical analysis

All data cleaning and preliminary analyses were conducted using R. A series of t-tests and Wilcoxon Signed Rank tests were conducted to assess sex differences on observed variables of interest. Subsequent structural equational modeling (SEM) pathway analyses were conducted using Mplus V.8. In the pathway analyses, all relationships were tested between latent factors, which are not directly observed, but constitute a measure that captures the common variance amongst the observed variables. Latent factors were used to capture more robust constructs that are free from measurement error. Each latent factor was specified within the model, for example the 12 items from the SCDC loading onto the SC difficulties latent construct. All latent factors were free to covary in each pathway model. All SA latent factor item residuals were specified to covary between time points (e.g. 7, 10 and 13 years old). All pathway analyses controlled for confounding variables, including IQ, SES and SA symptoms at age 7. All paths are reported as partial regression coefficients. In all pathway analyses, a robust maximum likelihood (MLR) estimator was used to account for the variables that deviate from normality. In addition, full information maximum likelihood (FIML) was used to estimate model parameters from raw data. Using FIML reduces the impact of bias from selective attrition by using all available data (Enders & Bandalos, 2001).

Previous research using ALSPAC data supports the construct validity of the DAWBA-SF and SCDC at 7, 10 and 13 years old (Pickard et al., 2017). Using Mplus, two confirmatory factor analyses were conducted to assess the construct validity of the Cambridge Hormones and Moods Project Friendship questionnaire and the Bullying and Friendship Interview Schedule. Firstly, a one-factor structure was specified for friendship quality with 5 indicators. Secondly, a two-factor structure was specified for peer victimisation, including overt victimisation with 5 indicators and relational victimisation with 4 indicators. Model fit was assessed using measures recommended for large datasets (Hooper, Coughlan, & Mullen, 2008). Absolute fit measures included the root mean square error of approximation (RMSEA; good fit < .05, acceptable fit < .08) and standardised root mean square residual (SRMR; good fit < .08). The comparative fit index (CFI; good fit > .95, acceptable fit > .90) was also used (Hu & Bentler, 1999; MacCallum, Browne, & Sugawara, 1996).

**Fig. 2:**
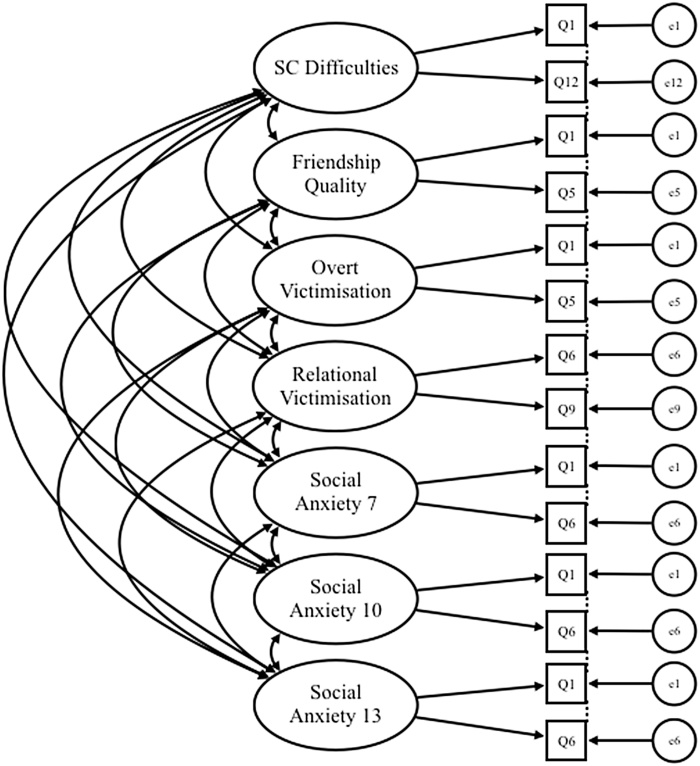
Illustration of saturated model with correlated latent factors, factor items and error variance. *Note.* SC = social and communication. Q = question. e = error.

A saturated measurement model was conducted to examine the associations between all latent factors (see Fig. 2). To test our hypothesis that SC difficulties lead to increased peer victimisation/poor friendship quality, we conducted a pathway model with SC difficulties as the predictor and friendship quality, overt victimisation and relational victimisation as the outcome variables. To test our mediation hypothesis, we conducted a multiple mediation pathway model to determine the extent to which the association between SC difficulties and SA symptoms at age 10 and 13 was mediated by overt victimisation and relational victimisation. We assessed the indirect effects of relational victimisation and overt victimisation on the pathway from SC difficulties to SA symptoms at 10 and 13 years old. Indirect effects were considered significant if the confidence intervals for the indirect effect did not include zero (Preacher & Hayes, 2004). To test our moderation hypothesis, we constructed an interaction pathway model with SC difficulties, friendship quality and their interaction term as predictors and SA symptoms at 10 and 13 years old as the outcome variables. In all pathway models, the effects of IQ, SES and SA symptoms at 7 years old were regressed out of SA at 10 and 13 years old. We applied a Bonferroni Correction to assess the significance of all pathway coefficients.

Model fit was tested using the Satorra-Bentler Scaled chi-square statistic (Satorra, 2000), which is used to compare chi-square when data are not normally distributed. To assess sex differences, likelihood ratio testing was employed to compare a baseline model with all paths freely estimated across sex to several nested models with paths equated across sex. To test sex differences in our interaction model, a baseline model was compared to a nested model where the interaction effect of SC difficulties and friendship quality was equated across sex. To test sex differences in our mediation model, a baseline model was compared to a nested model where the indirect effects of relational and overt victimisation was equated across sex.

## 3 Results

All questionnaire data were cleaned according to ALSPAC guidelines for data preparation (see Supplement 1). To test for selective attrition in our sample, we compared individuals who contributed SA data at age 7 and 13 years old (*n* = 5784) to those who only had available SA data at 7 years old (*n* = 2244). No significant difference was observed between individuals who had available SA data at all ages and those who only had data at 7 years old (*W* = 6540900, *p* =  .514).

Mean scores are presented in Table 1 for the total sample and separately for boys and girls. T-tests and Wilcoxon Signed-Rank tests with independent samples were conducted to assess sex differences in all variables of interest (see Table 1). Compared to girls, boys had significantly more SC difficulties and SA symptoms at age 7. Girls had significantly more SA symptoms at 13 years old, compared to boys. In addition, boys reported more overt victimisation, whereas girls reported more relational victimisation. No sex differences were observed for friendship quality and SA symptoms at 10 years old.

**Table 1:** Parent and child-reported characteristics on questionnaire data.

	Total (N = 8028)		Boys (n = 4130)		Girls (n = 3898)		Sex Differences	
Measures	n	Mean (SD) Range^a^	n	Mean (SD) Range^a^	n	Mean (SD) Range^a^	t-test/W^b^ *d/r*^b^	*p*
SC Difficulties_7_	8028	2.83 (3.71) 0-24	**4130**	**3.25 (4.14)** **0-24**	**3898**	**2.39 (3.13)** **0-24**	**7168900^b^** **−.1 ^b^**	**< .001**
Social Anxiety_7_	8028	0.87 (1.59) 0-12	**4130**	**0.95 (1.68)** **0-11**	**3898**	**0.79 (1.50)** **0-24**	**7677900^b^** **−.05 ^b^**	**< .001**
Social Anxiety_10_	6430	0.96 (1.68) 0-12	3262	1.00 (1.74) 0-12	3168	0.92 (1.62) 0-12	5061800 ^b^ −.02 ^b^	.10
Social Anxiety_13_	5784	1.22 (1.86) 0-12	**2906**	**1.11 (1.83)** **0-12**	**3168**	**1.32 (1.89)** **0-12**	**4486300^b^** **−.07 ^b^**	**< .001**
Friendship Quality_8_	5637	3.40 (2.38) 0-15	2830	3.48 (2.40) 0-15	2807	3.32 (2.36) 0-15	−2.56 .07	.01
Overt Victimisation_8_	5723	2.15 (2.47) 0-15	**2864**	**2.40 (2.59)** **0-15**	**2859**	**1.91 (2.32)** **0-14**	**3649200^b^** **−.1 ^b^**	**< .001**
Relational Victimisation_8_	5569	1.08 (1.78) 0-12	**2783**	**0.99 (1.73)** **0-12**	**2786**	**1.16 (1.82)** **0-12**	**4138800^b^** **−.07 ^b^**	**< .001**

*Note.* Means and standard deviations (SD) are reported for the total sample and split by gender. ^a^ Full range of scores in our sample. ^b^ Wilcoxon signed rank independent samples test statistic. SC = social and communication. Bold estimates indicate significance.

In the present sample, 26% of children (*n* = 1463) had experienced no victimisation, 40% of children (*n* = 2215) had experienced moderate victimisation (at least one type of victimisation experienced at an infrequent/frequent rate) and 34% of children (*n* = 1891) had experienced severe victimisation (at least two types of victimisation experienced at an infrequent/frequent rate).

### 3.1 Confirmatory factor analysis

Two models were specified to test the construct validity of the Cambridge Hormones and Moods Project Friendship questionnaire and the Bullying and Friendship Interview Schedule. All fit indices for the one-factor model with friendship quality at 8 years old (SRMR = .04, RMSEA [CI] = .084 [.074, .094], CFI = .91) were indicative of good/acceptable model fit. The results imply that the Cambridge Hormones and Moods Project Friendship questionnaire measures a coherent friendship construct. All fit indices for the two-factor model with peer victimisation at 8 years old (SRMR = .02, RMSEA [CI] = .019 [.014, .024], CFI = .99) were indicative of good model fit. The results imply that the Bullying and Friendship Interview Schedule measures two distinct constructs, including overt victimisation and relational victimisation.

### 3.2 Saturated model

A saturated model was conducted to examine the associations between all latent factors used in subsequent mediation and moderation pathway analyses (see Table 2).

**Table 2:** Correlation coefficients among all latent factors in the saturated model.

	R[CI]					
Measures	1	2	3	4	5	6
1 SC diffificulties_7_	1					
2 Social Anxiety_7_	**.21** **[.17, .24]**	1				
3 Social Anxiety_10_	**.21** **[.17, .25]**	**.53** **[.50, .57]**	1			
4 Social Anxiety_13_	**.17** **[.13, .20]**	**.40** **[.36, .43]**	**.51** **[.48, .54]**	1		
5 Friendship Quality_8_	**.15** **[.11, .19]**	.06 [.02, .10]	**.08** **[.04, .12]**	**.09** **[.05, .13]**	1	
6 Relational Victimisation_8_	**.09** **[.05, .13]**	.01 [-.03, .05]	.04 [-.00, .08]	**.08** **[.04, .12]**	**.31** **[.26, .36]**	1
7 Overt Victimisation_8_	**.21** **[.17, .25]**	.01 [-.03, .05]	.04 [.00, .09]	.03 [-.01, .07]	**.35** **[.31, .40]**	**.73** **[.69, .77]**

*Note.* Subscript numbers show the age at assessment. SC = social and communication. CI = 95% confidence intervals. Bold coefficients indicate significance.

### 3.3 Covariate path estimates

In all specified models, the effects of IQ, SES and SA symptoms at 7 years old were regressed out of the SA latent factor at age 10 (IQ: β = -.11, *p* < .001; SES: β = -.01, *p* =.45; SA_7_; β = .49, *p* <  .001) and 13 years (IQ: β = -.15, *p* < .001; SES: β = -.03, *p* = .05; SA_7_: β = .35, *p* < .001).

### 3.4 Do SC difficulties predict later peer victimisation/negative friendship quality?

Results from the pathway model analyses revealed that earlier SC difficulties significantly predicted negative friendship quality (β(se) = .15(.02), [95% CI] = [.11, .19], *p* < .001), increased overt victimisation (β(se) = .21(.02), [95% CI] = [.17, .25], *p* < .001) and relational victimisation (β(se) = .09(.02), [95% CI] = [.05, .13], *p* < .001), indicating a small to moderate effect of SC difficulties on later peer victimisation and friendship quality.

### 3.5 Does victimisation mediate the relationship between SC difficulties and SA symptoms?

**Fig. 3:**
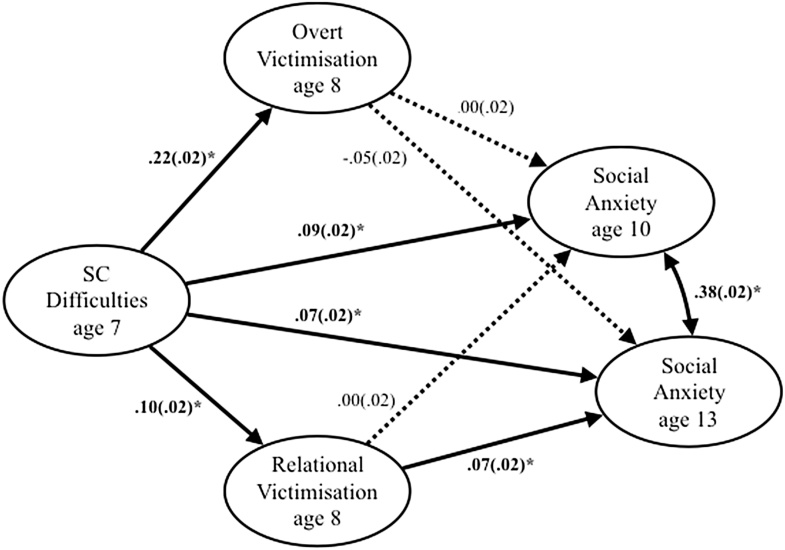
Mediation pathway model showing overt and relational victimisation as mediators in the developmental relationship from social and communication difficulties to social anxiety symptoms. *Note.* SC = social and communication. Standardized beta coefficients and standard errors are reported. All analyses controlled for IQ, SES and social anxiety symptoms at age 7. Bold paths indicate significance*.

Fig. 3 shows the standardised β coefficients for the multiple mediation pathway model testing whether overt and relational victimisation mediates the relationship between SC difficulties and SA at 10 and 13 years old. SC difficulties significantly predicted increased overt victimisation, relational victimisation and SA symptoms at all ages. No significant paths were observed from overt victimisation to SA at 10 or 13 years old. Relational victimisation significantly contributed to increased SA symptoms at 13, but not 10 years old. The indirect effects of SC difficulties via overt victimisation to SA at 10 (β(se) = .00(.00), [95% CI] = [-.01, .01]) and 13 (β(se) = -.01(.01), 95% CI = [-.02, -.00]) years old were not significant. Similarly, the indirect effects of SC difficulties via relational victimisation to SA at 10 (β(se) = .00(.00), [95% CI] = [-.00, .00]) and 13 (β(se) = .01(.00), [95% CI] = [.00, .01]) years old were not significant.

### 3.6 Does friendship quality moderate the relationship between SC difficulties and SA symptoms?

**Fig. 4:**
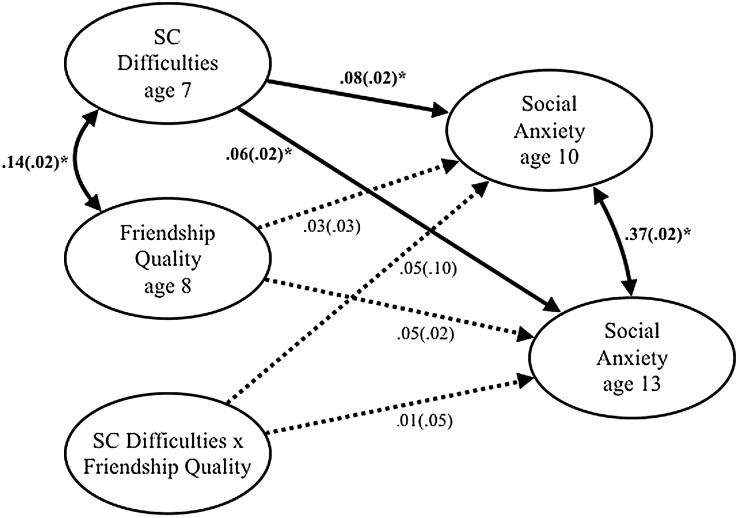
Moderation pathway model testing whether friendship quality moderates the effect of social and communication difficulties on social anxiety symptoms. *Note.* SC = social and communication. Standardized beta coefficients and standard errors are reported. All analyses controlled for IQ, SES and social anxiety symptoms at age 7. Bold paths indicate significance*.

Fig. 4 shows the standardised β coefficients for the pathway model testing the interaction between SC difficulties and friendships quality in predicting later SA symptoms at 10 and 13 years old. Our results show that SC difficulties significantly predict increased SA symptoms at all ages. Friendship quality did not predict increased SA symptoms at 10 and 13 years old. The interaction between SC difficulties and friendship quality was not a significant predictor of SA symptoms at 10 and 13 years old.

### 3.7 Sex differences

All pathway analyses are reported separately by sex (see Supplement 2). No sex differences were observed in our mediation model pathway analysis. In the mediation model, no significant decrease in model fit was observed when comparing a baseline model to a nested model where the indirect paths from SC difficulties to SA symptoms, via overt and relational victimisation, were constrained to be equal across boys and girls (Δχ^2^[df] = 0.62[6], *p* = .996). This suggests that no sex differences emerged in the indirect effects of overt and relational victimisation on later SA symptoms. In the moderation model, friendship quality significantly predicted later SA symptoms at 13 years old for boys (β(se) = .07 (.02), [95% CI] = [.02, .11], *p* < .01), but not girls (β(se) = .02 (.03), [95% CI] = [-.04, .08], *p* > .05), however, this difference was not significant (Δχ^2^[df] = 2.33[1], *p* = .127). Our results revealed that SC difficulties significantly predicted SA symptoms at 13 years old for girls (β(se) = .10 (.04), [95% CI] = [.03, .17], *p* < .01), but not boys (β(se) = .06 (.03), [95% CI] = [.00, .12], *p* > .01), however, this difference was not significant (Δχ^2^[df] = 2.05 [1], *p* = .394). Furthermore, in the moderation model, no significant decrease in model fit was observed when comparing a baseline model to a nested model which constrained all interaction paths to be equal across boys and girls (Δχ^2^[df] = 0.74[3], *p* = .863). This suggests that no sex differences are observed in the interaction between SC difficulties and friendship quality predicting later SA symptoms.

## 4 Discussion

In a large population-based longitudinal sample, we used both child-reported and parent-reported data to identify whether friendship quality, and overt and relational peer victimisation are mechanisms that influence the developmental pathway from SC difficulties to SA symptoms in childhood. First, supporting and extending previous literature, we found prospective associations between parent-reported SC difficulties and increased self-reported negative friendship quality, and overt and relational victimisation. Second, the data partially supported our second hypothesis, showing that relational victimisation, but not overt victimisation, made a small contribution towards parent-reported SA symptoms at 13 years old. However, friendship quality did not predict increased SA symptoms in later childhood. Our results did not support our mediation and moderation hypotheses. Friendship quality did not moderate the developmental relationship between SC difficulties and later SA symptoms, such that having negative friendship qualities did not place children with SC difficulties at an increased risk of developing SA in later childhood. Additionally, we found no evidence for our mediation hypothesis, suggesting that the developmental relationship between SC difficulties and SA symptoms was not mediated by increased relational or overt victimisation. In addition, no sex differences were observed in our moderation or mediation analyses. Although our findings reflect some consistent evidence with previous research, our results do not support two of our prior predictions, suggesting that peer victimisation and friendship quality do not influence the pathway from SC difficulties to SA in childhood. In the following sections, we will discuss our findings in relation to previous research, as well as considering limitations, implications and avenues for future research.

In accordance with previous research in children and adolescents (Cappadocia et al., 2012; Crawford & Manassis, 2011; Fox & Boulton, 2006), we found evidence for associations between SC difficulties, friendship quality and peer victimisation in a large population-based sample of children. The magnitude of these associations are comparable to previous research with anxious and non-anxious children (Crawford & Manassis, 2011; Fox & Boulton, 2006). Extending previous research, SC difficulties were shown to predict an increase in peer victimisation and negative friendship quality throughout childhood. These novel findings emphasise that children with SC difficulties as young as 7 years old are at an increased risk of experiencing adverse peer problems. Our findings support the dimensional view of psychopathology, highlighting that children with varying degrees of SC difficulties, including those with only modest difficulties, are at an increased risk of developing poor quality friendships and experiencing increased victimisation. Our findings emphasise the need for preventative interventions that target peer problems for children with SC difficulties who may be at the most immediate risk.

Negative friendship quality and peer victimisation, in particular relational victimisation, are linked with increased SA symptoms in both clinical and subclinical youth populations (Erath et al., 2007; Ginsburg et al., 1998; La Greca & Lopez, 1998; Greco & Morris, 2005; La Greca & Harrison, 2005; Storch, Masia-Warner, Crisp, & Klein, 2005). In accordance, our novel longitudinal findings using a large population-based sample show small associations between childhood friendship quality and later SA symptoms, as well as an association between increased relational victimisation and greater SA symptoms at 13 years old. These findings are in accord with etiological models of SA, in both normative development and neurodevelopmental disorders (Bellini, 2006; Spence & Rapee, 2016), which propose that experiences of negative peer relationships contribute towards the development of SA in childhood. Partially supporting our second hypothesis, we found that children who experienced relational victimisation were at an increased risk for SA at age 13 years. Consistent with research in adolescents (Dempsey et al., 2009; Siegel et al., 2009; Storch & Masia-Warner, 2004; Storch et al., 2005), these findings indicate that relational victimisation, but not overt victimisation, may act as an independent risk factor for SA in pre-adolescent children. Experiencing peer victimisation, specifically relational victimisation (e.g. spreading rumours and excluding peers), may increase a child’s negative self-evaluations and subsequent avoidance and withdrawal from negative social interactions, which together can exacerbate feelings of SA. Further longitudinal research is needed to extend our understanding of the role of relational victimisation in the development of SA in childhood.

Interestingly, our moderation results provide new information regarding putative mechanisms that influence the developmental pathway from SC difficulties to SA symptoms (Pickard et al., 2017). Our findings show that experiencing negative friendship qualities does not exacerbate the risk of a child with increased SC difficulties, developing SA. Whilst in contrast to our prior predictions, this finding contributes toward our knowledge of the complex etiological pathways and influential mechanisms that increase and/or decrease a child with SC difficulties' risk of developing SA. Equally, these findings may be indicative of age-related differences in the impact of friendships on SA, such that friendships acquired later in development may act independently or in conjunction with other risk factors to predict SA in late adolescence. The increasing impact of friendships on SA later in development, may stem from increased peer influences, pressures to retain friends and deal with teasing, and the expansion of important peer networks (La Greca & Harrison, 2005; Spence & Rapee, 2016). This is supported by extensive research reporting associations between SA and friendships in adolescence (Erath et al., 2007; La Greca & Lopez, 1998; La Greca & Harrison, 2005). Although our moderation hypothesis was not supported, it should be noted that the impact of friendship quality on SA may be exacerbated across development, as greater social pressures and challenges emerge throughout adolescence. Our findings highlight the need for longitudinal research assessing the relationship between SC difficulties, aspects of friendships, and SA across development. Future research will be important for elucidating the age-related differential impact of SC difficulties and friendship quality, if any, in predicting SA across childhood and adolescence.

In contrast to prior models (Spence & Rapee, 2016), our findings provide evidence to suggest that the developmental relationship from SC difficulties to SA symptoms was not explained by increased overt or relational victimisation in childhood. Similar cross-sectional findings have been reported in a sample of non-anxious children, with both SC difficulties and victimisation severity not predicting anxiety symptoms. However, these relationships were observed in the anxious children (Crawford & Manassis, 2011). Hence, victimisation may have a significant impact on anxiety levels when experienced alongside clinical symptomatology, such as severe SC difficulties. In accordance, young people with ASD, who lie at the extreme end of the SC ability continuum, experience both increased victimisation and high rates of SA (Cappadocia et al., 2012; Salazar et al., 2015), compared to their peers without ASD. Future research could test these predictions by exploring our predictions in children with high subclinical or clinical levels of SC difficulties.

It could be postulated that victimisation experienced during childhood versus adolescence may have a differential impact on the development of SA. Adolescence is characterised as a sensitive period where rapid social and emotional changes occur, such as increasing peer pressures and social demands outside of the family home. In addition, for children in the UK the school environment and subsequent peer group often changes between 10 and 13 years old. Hence, there may be a delayed effect of victimisation in childhood, which due to increased social demands and changes in social circles has a greater impact on SA during adolescence. As such, many of the expected relationships in the current study may have been present if this research had been carried out in adolescence, where experiences of peer pressure and the need to fit in amongst their social group becomes salient. In line with this, a recent meta-analysis of peer victimisation studies found that the association between relational victimisation, sadness and anxiety strengthens across development (Casper & Card, 2017). This is reflected in our results, as relational victimisation predicted SA symptoms at 13, but not 10, years old. This etiological pathway may be particularly important for adolescents with SC difficulties, who are already at a heightened risk of being victimised by their peers. Although this hypothesis is not directly testable in the present study, longitudinal research tracking the developmental impact of peer victimisation on SA for individuals who experience varying degrees of SC difficulties is needed. Research of this nature will shed light on the impact of developmentally specific risk factors of SA, ultimately informing the design and implementation of effective treatments for SA in younger populations.

We observed small sex differences in overt and relational victimisation severity, SC difficulties and SA at 13 years old. In line with previous literature, girls reported higher rates of relational victimisation, whereas boys reported higher rates of overt victimisation (La Greca & Lopez, 1998; Greco & Morris, 2005; Ranta et al., 2013). Furthermore, parent-reports show that boys experienced greater SC difficulties, whilst girls experienced increased SA symptoms at 13 years old, consistent with epidemiological findings (Beidel et al., 2007). No sex differences were observed in our mediation and moderation analyses, indicating that friendship quality and peer victimisation do not differentially influence the pathway from SC difficulties to SA symptoms for boys and girls. Studies examining sex differences in the mechanisms that contribute towards SA across childhood and adolescence is warranted, as important differences may emerge throughout development.

The present study is strengthened by the longitudinal design that employed a large population-based sample of children to explore novel developmental relationships. However, our findings should be considered in light of the following limitations. Firstly, our analyses relied on a mixture of parent-report and child-report measures. Furthermore, data for child-report measures of friendship quality and victimisation were only available at 8 years old, limiting the generalisability of our findings beyond this specific age group. Additionally, these data were collected from young children and it should be noted that different results might be observed if parent-reported or peer-reported data were used and if these relationships were investigated in adolescence. Furthermore, several of the measures rely on a small number of items that may not be able to capture the full breadth of the construct under investigation. Therefore, future longitudinal research incorporating richer multi-informant measures at multiple time points would be beneficial in order to assess the predictions outlined in this study.

### 4.1 Conclusion

In conclusion, our novel longitudinal findings demonstrate that experiencing peer victimisation and negative friendship quality in early childhood does not increase a child with SC difficulties risk of developing SA in later childhood. These findings have important implications for our current etiological models of SA (Spence & Rapee, 2016), suggesting that further longitudinal research exploring the complex causal pathways, including mediating and moderating risk factors, across development is required to gain a deeper understanding of the age-specific risk factors contributing towards SA in childhood and adolescence. Several studies support the role of negative friendships and peer victimisation in the development of several mental health problems (Reijntjes, Kamphuis, Prinzie, & Telch, 2010). We propose that research employing longitudinal designs is necessary to explore and identify mechanisms that help explain the developmental relationship between SC difficulties and SA. This research will help to inform effective, developmentally specific, treatments that apply individualistic approaches to target the risk factors contributing towards a child's pathway from SC difficulties to SA.

## Declaration of interest

None.

## Fundings

The UK Medical Research Council and Wellcome (Grant ref: 102215/2/13/2) and the University of Bristol provide core support for ALSPAC. Miss H. Pickard is funded by the Medical Research Council (MRC) and Autistica. This publication is the work of the authors, who will serve as guarantors for the contents of this paper.
