# Supplementary material for: Navigating the social world: The role of social competence, peer victimisation and friendship quality in the development of social anxiety in childhood
Source: J Anxiety Disord. 2018 Dec;60:1–10. doi: 10.1016/j.janxdis.2018.09.002 (PMC6269163; doi:10.1016/j.janxdis.2018.09.002)
Supplement: Supplementary file 1 [file mmc1.docx]

**Navigating the social world: the role of social competence, peer victimisation and friendship quality in the development of social anxiety in childhood.**

Supplementary Material 1 – Data preparation

In accordance with Avon Longitudinal Study of Parents and Children (ALSPAC) documentation for handling data, all variables were recoded and total scores were created using the following prorating system. Any individuals with more than 50% missing data on the Social and Communication and Disorder Checklist (SCDC; > 7 item) at age 7 years and the Development and Wellbeing Assessment – Social Fears subscale at age 7, 10 or 13 years were marked as missing data and were not included in any further analysis. Furthermore, individuals with missing data or who gave one response of ‘don’t know’ on one or more items on the Cambridge and Hormones Friendship Questionnaire were marked as missing data on the total score. In addition, children with two or more missing items on the relational subscale or overt subscale of the Bullying and Friendship Interview Schedule were marked as missing data. We performed a series of checks for outliers and normality. No outliers were identified. Skewness and kurtosis values showed that several of our total score variables were non-normally distributed. In these cases, non-parametric tests were used. The final sample consisted of parent and child reported data from 8,028 children at age 7, 8, 10 and 13 years old.
