# Supplementary material for: Navigating the social world: The role of social competence, peer victimisation and friendship quality in the development of social anxiety in childhood
Source: J Anxiety Disord. 2018 Dec;60:1–10. doi: 10.1016/j.janxdis.2018.09.002 (PMC6269163; doi:10.1016/j.janxdis.2018.09.002)
Supplement: Supplementary file 2 [file mmc2.docx]

**Navigating the social world: the role of social competence, peer victimisation and friendship quality in the development of social anxiety in childhood.**

Supplementary Material 2 – Pathway analyses split by sex

**Table S1** Correlation coefficients among all latent factors in the saturated model split by sex

|  |  |  |  | R[CI] |  |  |  |
| --- | --- | --- | --- | --- | --- | --- | --- |
| Measures | 1 | 2 | 3 | 4 | 5 | 6 | 7 |
| 1 SC diffificulties_7_ | 1 | **.18**  **[.13-.23]** | **.21**  **[.16-.26]** | **.17**  **[.13-.22]** | **.18**  **[.12-.24]** | **.09**  **[.03-.14]** | **.18**  **[.12-23]** |
| 2 Social Anxiety_7_ | **.22**  **[.17-.27]** | 1 | **.50**  **[.46-.55]** | **.35**  **[.30-.40]** | **.09**  **[.03-.13]** | .02  [-.04-.07] | .02  [-.04-.07] |
| 3 Social Anxiety_10_ | **.22**  **[.17-.26]** | **.55**  **[.51-.60]** | 1 | **.51**  **[.46-.56]** | **.10**  **[.04-.16]** | .04  [-.01-.10] | .05  [-.01-.11] |
| 4 Social Anxiety_13_ | **.18**  **[.13-.24]** | **.43**  **[.38-.48]** | **.51**  **[.46-.56]** | 1 | .08  [.02-.13] | **.10**  **[.04-16]** | .06  [-.00-.11] |
| 5 Friendship Quality_8_ | **.13**  **[.07-.18]** | .03  [-.02-.08] | .05  [-.01-.10] | **.11**  **[.05-.16]** | 1 | **.34**  **[.27-.41]** | **.37**  **[.30-.44]** |
| 6 Relational Victimisation_8_ | **.11**  **[.05-.17]** | .01  [-.04-.07] | .03  [-.03-.09] | .05  [-.01-.11] | **.28**  **[.20-.36]** | 1 | **.74**  **[.68-.80]** |
| 7 Overt Victimisation_8_ | **.21**  **[15-26]** | -.02  [-.07-.04] | .03  [-.03-.09] | .02  [-.04-.07] | **.34**  **[.28-.41]** | **.74**  **[.68-.80]** | 1 |

*Note.* Subscript numbers show the age at assessment. SC = social and communication. CI = 95% confidence intervals. Bold coefficients indicate significance. Correlation coefficients for boys are reported below the diagonal line.


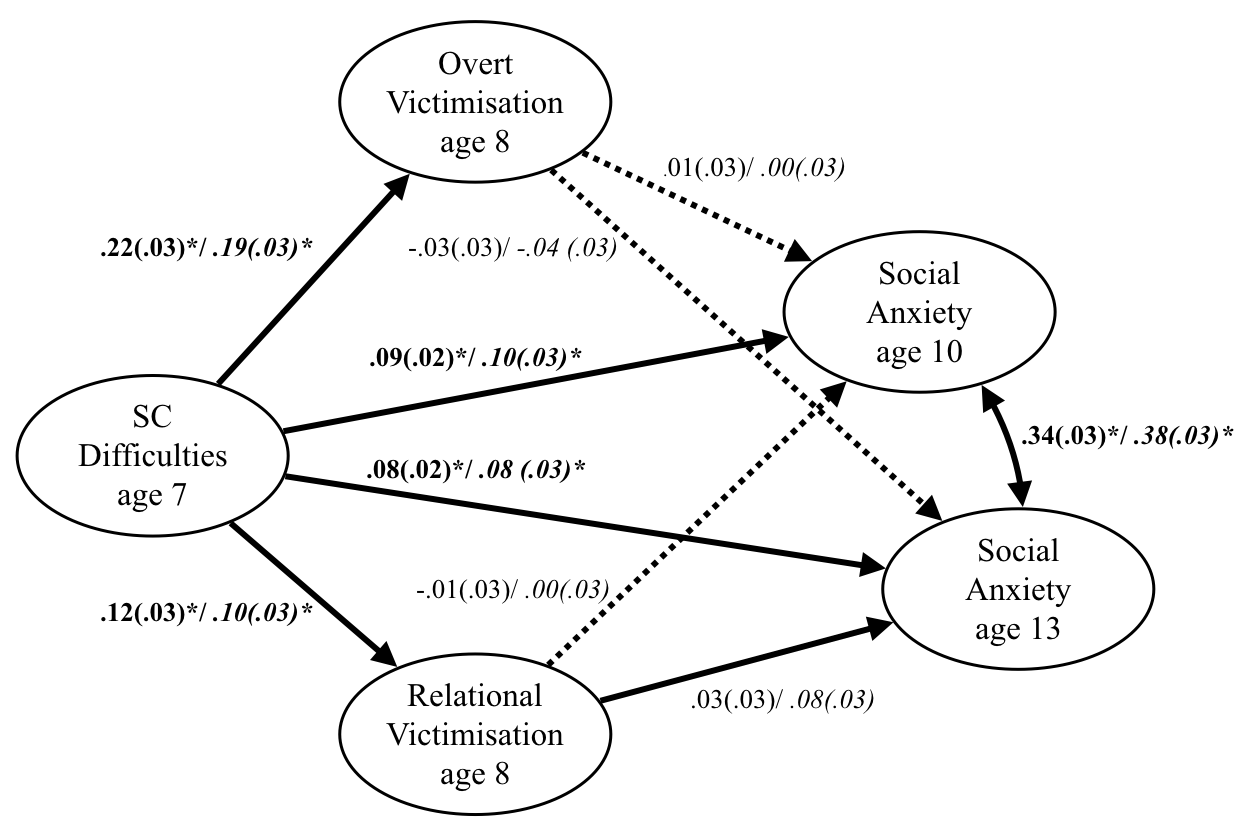


**Figure S1** Mediation pathway model showing overt and relational victimisation as mediators in the developmental relationship from social and communication difficulties to social anxiety symptoms split by sex

*Note.* SC = social and communication. Standardized beta coefficients and standard errors are reported (boys / *girls*). All analyses controlled for IQ, SES and social anxiety symptoms at age 7. Bold paths indicate significance**.*


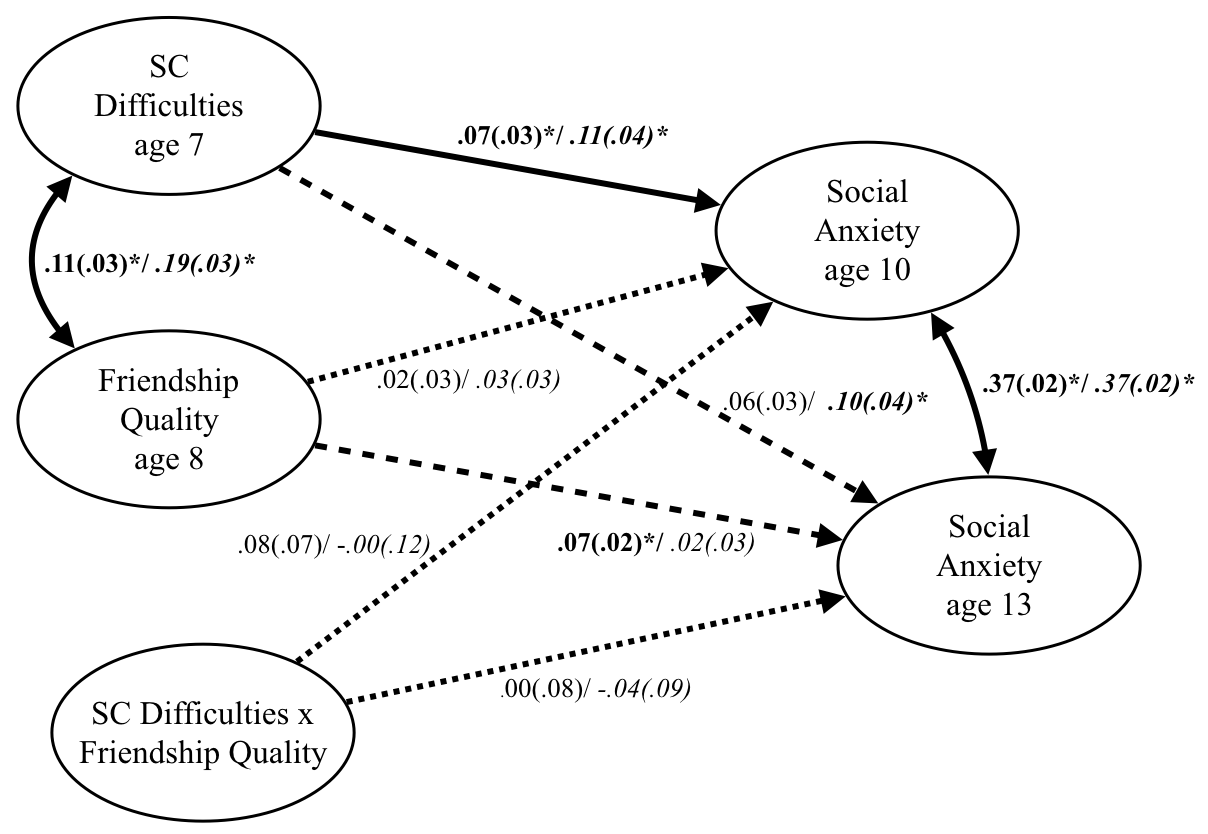


**Figure S2** Moderation pathway model testing whether friendship quality moderates the effect of social and communication difficulties on social anxiety symptoms split by sex

*Note.* SC = social and communication. Standardized beta coefficients and standard errors are reported (boys / *girls*). All analyses controlled for IQ, SES and social anxiety symptoms at age 7. Bold paths indicate significance*.
